# Relevance of Peptide Homeostasis in Metabolic Retinal Degenerative Disorders: Curative Potential in Genetically Modified Mice

**DOI:** 10.3389/fphar.2021.808315

**Published:** 2022-01-13

**Authors:** Etelka Pöstyéni, Alma Ganczer, Andrea Kovács-Valasek, Robert Gabriel

**Affiliations:** ^1^ Department of Experimental Zoology and Neurobiology, University of Pécs, Pécs, Hungary; ^2^ János Szentágothai Research Centre, University of Pécs, Pécs, Hungary

**Keywords:** diabetic retinopathy, ischemia, neuropeptides, apoptosis, oxidative stress, inflammation

## Abstract

The mammalian retina contains approximately 30 neuropeptides that are synthetized by different neuronal cell populations, glia, and the pigmented epithelium. The presence of these neuropeptides leaves a mark on normal retinal molecular processes and physiology, and they are also crucial in fighting various pathologies (e.g., diabetic retinopathy, ischemia, age-related pathologies, glaucoma) because of their protective abilities. Retinal pathologies of different origin (metabolic, genetic) are extensively investigated by genetically manipulated *in vivo* mouse models that help us gain a better understanding of the molecular background of these pathomechanisms. These models offer opportunities to manipulate gene expression in different cell types to help reveal their roles in the preservation of retinal health or identify malfunction during diseases. In order to assess the current status of transgenic technologies available, we have conducted a literature survey focused on retinal disorders of metabolic origin, zooming in on the role of retinal neuropeptides in diabetic retinopathy and ischemia. First, we identified those neuropeptides that are most relevant to retinal pathologies in humans and the two clinically most relevant models, mice and rats. Then we continued our analysis with metabolic disorders, examining neuropeptide-related pathways leading to systemic or cellular damage and rescue. Last but not least, we reviewed the available literature on genetically modified mouse strains to understand how the manipulation of a single element of any given pathway (e.g., signal molecules, receptors, intracellular signaling pathways) could lead either to the worsening of disease conditions or, more frequently, to substantial improvements in retinal health. Most attention was given to studies which reported successful intervention against specific disorders. For these experiments, a detailed evaluation will be given and the possible role of converging intracellular pathways will be discussed. Using these converging intracellular pathways, curative effects of peptides could potentially be utilized in fighting metabolic retinal disorders.

## 1 Introduction

Neuropeptides (NPs) are essential for maintaining a healthy nervous system and are present in many forms and sizes. They act as neurotransmitters, neuromodulators, immunomodulators and play a non-negligible role in endocrine regulation (Burbach, 2011). NPs are produced by neuronal and non-neuronal cells alike and are also expressed in abundance by various cell types of the retina (Bagnoli et al., 2003).

The mammalian retina is a multi-layered, light-sensitive organ of neural origins and contains five major classes of neurons, namely the photoreceptors (PR), horizontal cells (HC), bipolar cells (BC), amacrine cells (AC) and retinal ganglion cells (RGC) which all play a role in the generation of the visual signal. Homeostasis in the retina is also complemented by support from glial cells (most importantly the Müller glia—MC, microglia and astrocytes) and epithelial cells, which form the retinal pigment epithelium (RPE). MCs and the RPE simultaneously contribute significantly to NP production and they are known to express multiple types of NPs while retinal neurons (mainly ACs) tend to always express a singular type (for a summary, see Table 1 but please note that peptides are represented in alphabetic order and not in order of relevance). Besides the glial and epithelial support, healthy vascularization is also crucial for maintaining normal retinal function. The neurovascular unit, which encompasses all neuronal, glial and vascular elements that exist in close interdependence, is an especially vulnerable target for pathologies affecting retinal integrity (Nian et al., 2021). Retinal diseases are the leading cause of blindness worldwide and can be divided into two major categories based on their origin: genetic or metabolic. Both of these pose a substantial threat to retinal health and vision.

**TABLE 1:** Peptides and proteins markedly relevant for retinal health in the mouse, rat and human retinas.

Row	Peptides and other factors markedly relevant for retinal health in the mouse, rat and human retinas^a^					
	Peptides	Abbreviation	Source	Receptors	Receptor expression	Main function
1	Angiotensin II	Ang II	PR^3^, (GC)^3^, MC^3^ (Senanayake et al., 2007) + extraretinal	AT1R (vasoconstrictor), AT2R (vasodilatator)	AT1R: RPE^1,3^, PR^1,3^, ON BC^2^, AC^1,2,3^, GC^1,3^, MC^2,3^, astrocyte^2^	Regulation of blood pressure
					AT2R: RPE^1,3^, AC^1,2^, GC^1,2,3^, MC^3^ (Choudhary et al., 2017; Verma et al., 2019)	
2	Angiotensin (1-7)	Ang (1-7)	MC^3^ (Senanayake et al., 2007)	MasR	PR^1^, GC^1^, MC^1^ (Prasad et al., 2014)	Counteracts Ang II
3	Erythropoietin	EPO	RPE^3^, neuroretina^3^, + extraretinal (Hernández and Simó, 2012; Luo et al., 2015) + extraretinal	EPOR	GC^1,3^ (Shah et al., 2009)	Hormone, cytokine, red blood cell production
4	Glucagon-like Peptide-1	GLP-1	Extraretinal	GLP-1R	MC^2^, INL^2^, GCL^2,3^ (Zhang et al., 2009; Zhang et al., 2011; Hebsgaard et al., 2018)	Glucose dependent insulin secretion, inhibits glucagon secretion
5	Neuropeptide Y	NPY	AC^1,2,3^, dAC^1,2,3^ (Sinclair and Nirenberg, 2001; Christiansen et al., 2018a)	Y1, Y2, Y4, Y5	RPE^2,3^, PR^2^, BC^2^, HC^2^, AC^2^, GC^2^, MC^2,3^, microglia^2^ (Santos-Carvalho et al., 2015)	Retina development, neuromodulator
6	Pituitary adenylate cyclase-activating polypeptide	PACAP	HC^2^, AC^2^, (GC)^2^ (Izumi et al., 2000)	PAC1R, VPAC1R, VPAC2R	PAC1R: AC^2^, GC^2^ (Dénes et al., 2014)	Neuromodulator
7	Somatostatin	SST	RPE^3^, AC^1,2^, dAC^1,2^, neuroretina^3^ (Cristiani et al., 2002; Dubovy et al., 2017)	sst1-5	sst_2A_: rodBC^1,2^, HC^1,2^, AC^1,2^	Hormone, neurotransmitter, neuromodulator
					sst_4_: GC^1^ (Cristiani et al., 2002)	
8	Substance P	SP	BC^1^, AC^1,2,3^, dAC^3^, GC^3^ (Cuenca et al., 1995; Casini et al., 2000; Catalani et al., 2004)	NK1, (NK3)	NK1: ON BC^1^, AC^1,2^, dAC^2^, GC^2^	Neurotransmitter, neuromodulator
					NK3: OFF BC^1,2^, AC^2^ (Casini et al., 2000; Catalani et al., 2004)	

Row	Other factors	Abbreviation	Source		Receptor expression	Main function
9	Brain-derived neurotrophic factor	BDNF	AC^1,2^, (GC)^1,2^, MC^3^ (Bennett et al., 1999; Oku et al., 2002; Vecino et al., 2002; Avwenagha et al., 2006)	TrkB	PR^3^, AC^1,2^, GC^1,2^ (Cellerino and Kohler, 1997; Vecino et al., 1998; Vecino et al., 2002)	Neuron growth, differentation and survival, synaptogenesis
10	Pigment epithetlium derived factor	PEDF	RPE (Rebustini et al., 2021)	PEDF-R	RPE, PR (Rebustini et al., 2021)	Neurothrophic, anti-angiogenic, anti-tumorigenic
11	Vascular endothelial growth factor	VEGF	RPE^2^, PR^3^, HC^3^, BC^3^, AC^3^, (GC)^3^, MC^3^ (Sall et al., 2004; Saint-Geniez et al., 2008)	VEGFR1, VEGFR2, VEGFR3	VRGF2R: RPE^2,3^, PR, MC (Famiglietti et al., 2003; Saint-Geniez et al., 2008)	Angiogenesis

A variety of neuropeptides and other proteins are expressed in the mammalian retina, along with their receptors, and these molecules play a diverse role in maintaining normal retinal function. Amacrine cells ACs, müller cells and the cells of the pigment epithelium contribute the most significantly to intraretinal neuropeptide production. Ganglion cells are represented in brackets as the peptides and proteins released from their axons exert their effects outside of the retina. Abbreviations: AC, amacrine cell; BC, bipolar cell; dAC, displaced amacrine cell; GC, ganglion cell; GCL, ganglion cell layer; HC, horizontal cell; INL, inner nuclear layer; IPL, inner plexiform layer; MC, Müller cell; PR, photoreceptor; RPE, retinal pigment epithelium.

a Peptides and proteins contained in this table are listed in alphabetical order and not in any order of importance or other relevance: ^1^mouse, ^2^rat, ^3^human.

In the last few decades, the advancement of technology has opened the way for a new era in scientific research. The development of genetically modified (GM) animal models, in particular, has led to a wealth of knowledge concerning the genetic background and the genotype-phenotype connection of disease pathogenesis at molecular level. Mice, for example, have proven to be especially useful models in this regard. These transgenic models also allow researchers to test novel and innovative therapeutic approaches, bringing us once again closer to fully understanding disease etiology. One notable field of research where transgenic animal models have proven to be truly effective is the mimicking of typical human retinal pathologies with genetic origin; the MGI (Mouse Genome Informatics) database currently contains 27 different transgenic models which can be used as a model for multiple retinal diseases (e.g., retinitis pigmentosa, age-related macular degeneration, retinoblastoma, Stargardt disease, cone-rod dystrophy) (for an extensive summary, see Supplementary Table S1). The development of animal models of hereditary retinal diseases is based, first and foremost, on the identification of disease causing genes, which can then later be removed or (re)introduced to the genome of the model animal. Results from such experiments have already been extremely useful in answering fundamental questions regarding both disease pathophysiology and normal gene function (Flannery, 1999; Haruyama et al., 2009; Maeda and Maeda, 2018).

Diseases of metabolic origin (e.g., diabetic retinopathy, damage caused by ischemia or complications related to aging), however, have proven to be more challenging to replicate with transgenic models as their genetic background is significantly more complex and their pathophysiology is affected by a combination of (often unmapped) genetic and environmental factors (Ramkumar et al., 2010; Fletcher et al., 2011).

In the last 50 years, the mouse retina has been the subject of extensive research. Genetically modified mouse strains have served on the forefront of transgenic research (Jaenisch and Mintz, 1974) and have been frequently utilized ever since (Navabpour et al., 2020). In general, the mouse can be considered a well-known and acknowledged model of the mammalian retina, along with the rat (Ellenbroek and Youn, 2016). Rats, while not typically applied as transgenic models, have played a crucial role in physiological and biomedical research (Smith et al., 2019), yielding numerous non-GM models mimicking human pathologies (Szpirer, 2020). Because of that, we chose to build the current review first and foremost on the research carried out in mice, supported by data obtained from rat models and, whenever possible, complemented by information regarding the workings of the human retina.

The aim of the present study, therefore, has been to provide a summary on the current status of genetic mouse models utilized in the research of retinal NPs, with particular attention paid to the utilization of said models in studying major retinal diseases of metabolic origin. To achieve this, we will i) discuss the current understanding of NPs expressed in the mammalian retina, ii) the roles they play in enhancing or alleviating disease pathophysiology in major retinal diseases of metabolic origin (namely diabetic retinopathy, ischemia), and iii) highlight intracellular pathways where transgenic modifications connected to NP expression can offer us further insight to combat retinal degeneration. More specifically, we highlight how neuropeptide-related research embedded into different knockout and transgenic mouse models can help us answer a plethora of previously unanswered questions about complex manifestations of metabolic retinal diseases and their new promising therapeutic opportunities.

## 2 Neuropeptides Most Relevant for Retinal Health

In the present day, NPs are generally defined as biologically active substances that are produced by neurons in order to modulate nervous system functions. While the precise term “neuropeptide” has only been coined by David de Wied in 1971 (de Wied, 2000; Burbach, 2011), signal molecules of this kind have been the focus of extensive study since the early days of endocrinology, as many of them had first been discovered as part of the hormonal regulatory system (Burbach, 2011). The diversity in origin (neuronal or non-neuronal) and function (neurotransmitter, neuromodulator or an agent of endocrine, paracrine or autocrine regulation) has provided a substantial material for research. Beside the classical molecules produced by nerve cells, putative NPs (produced by glial cells, for example) also play an important role in maintaining a healthy and functioning nervous system (Burbach, 2011). NPs are (both in the classical and putative sense) also expressed in the retina (Bagnoli et al., 2003) by both neurons, glia and cells of the RPE (Table 1). A variety of NPs have been demonstrated to play an important and diverse role in maintaining healthy retinal function and therefore could already be used to prevent tissue and cell damage when tissue integrity is compromised as a result of pathological processes (Otani et al., 2000; Junk et al., 2002; Zhang et al., 2011). While there are many questions still waiting to be answered, our current knowledge regarding NPs far exceeds the boundaries of this article. As such, this paper focuses specifically on peptides with substantial influence on retinal health. For a short summary of the selected peptides discussed in detail, please refer to Table 1.

### 2.1 The Retinal Renin-Angiotensin System

The renin-angiotensin system (RAS) plays an essential role in regulating blood pressure and keeping fluid volume and electrolyte composition under control. Currently, the existence of a local RAS in the retina seems well supported (see row 3 of Table 1), considering that its individual components (hormonal and enzymatic alike) appear to be all expressed in retinal tissue (Choudhary et al., 2016).

The active form of angiotensin, angiotensin II (Ang II), is generated by the cleavage of angiotensinogen into angiotensin I in the presence of renin, which in turn is cleaved by the angiotensin converting enzyme (ACE) to form Ang II. The release of Ang II is generally known to induce vasoconstriction, thirst and the release of aldosterone (Culman et al., 1995) and has also been demonstrated to possess pro-inflammatory effects (Ruiz-Ortega et al., 2001; Chen et al., 2006; Ruiz-Ortega et al., 2006; Wilkinson-Berka et al., 2019), which are all conveyed through the angiotensin II type 1 receptor (AT1R; Forrester et al., 2018). AT1 receptors are G-protein coupled receptors that ultimately trigger the release of Ca^2+^ from the endoplasmic reticulum which results in the activation of protein kinase C (PKC) and additional kinase enzyme activation in the extracellular signal-regulated kinase (ERK1/2) pathway (Simões et al., 2020). In contrast, the type 2 receptor (AT2R) is generally understood to act against AT1R, as it is known to elicit vasodilation through the generation of nitric oxide (NO) and contribute to cell survival (Reinecke et al., 2003; Carey, 2017; Verma et al., 2019). The signaling pathways connected to AT2R, however, are not yet fully understood (Sadybekov and Katritch, 2020). It is believed that AT2R activation does not follow the traditional G-protein coupled receptor mechanisms and affects downstream mediators instead of directly altering cyclic adenosine monophosphate (cAMP) or Ca^2+^ levels. It has been proposed that receptor activation causes ERK1/2 dephosphorylation (Porrello et al., 2009), counteracting the effects of AT1R. Connections between AT2R activation and an increase in NO and cyclic guanosine monophosphate (cGMP) production have also been observed (Porrello et al., 2009). The AT1R is expressed by a large variety of retinal cells (RPE, PR, AC, GC, MC and certain ON BC) in the human, rat and mouse retinas (Senanayake et al., 2007; Choudhary et al., 2016), while AT2R expression is less widespread and not expressed by PR and BC in mice (Verma et al., 2019) (for comparison, see row 1 of Table 1).

Besides Ang II, other derivatives of Ang I also exist: angiotensin-(1-7) or Ang (1-7) is a peptide generated by angiotensin converting enzyme 2 (ACE2), which also plays an important role in counteracting the effects of Ang II and therefore has been studied for its protective properties (Santos et al., 2018). The effects of Ang (1-7) (including anti-inflammatory and antiproliferative effects, antioxidant properties and vasodilation through NO release) are conveyed through the Mas receptor, forming the ACE2/Ang (1-7)/Mas protective axis (Fletcher et al., 2010; Peiró et al., 2013; Prasad et al., 2014). The Mas receptor so far has been connected to adenylate cyclase-related signal pathways (through Gαi2), with receptor activation having negative impact on cAMP levels and ERK1/2 phosphorylation (Burghi et al., 2017). Ang (1-7) and its receptor are also expressed in the retina (see row 2 of Table 1) (Senanayake et al., 2007; Verma et al., 2012; Prasad et al., 2014). Ang (1-7) is present in the MCs of the human retina (Senanayake et al., 2007) and the mouse retina contains the Mas receptor on PRs, GCs and MCs (Prasad et al., 2014).

### 2.2 Erythropoietin

Erythropoietin (EPO) is a glycoprotein hormone primarily produced by the kidneys under hypoxia and its main function is the stimulation of erythropoiesis. However, EPO production has been demonstrated in various tissues beside the kidneys, including the retina, where the hormone is suspected to play an important role during retinal development (Reid and Lois, 2017). The EPO receptor (EPOR) is expressed in both nuclear layers in addition to its expression in the GCL and the RPE of the human retina, and human and mouse GCs are both known to express EPOR (Hernández and Simó, 2012; Luo et al., 2015; Shah et al., 2009; see row 3 of Table 1). Ligand binding of the EPO receptor is known to trigger dimerization, although the pathways associated with the erythropoietic and neuroprotective functions of EPO seem to differ to some degree (Sollinger et al., 2017). In the first step of the signaling pathway, the dimerization of the EPOR activates the janus kinase 2 (JAK2) enzyme, which is also present in neurons (Digicaylioglu and Lipton, 2001). The neuroprotective capabilities of EPO appear to be conveyed through nuclear factor kappa B (NF-κB), mitogen-activated protein kinase (MAPK), signal transducer and activator of transcription 5 (STAT5) and phosphoinositide 3-kinase (PI3K) activation (Ma et al., 2016; Sollinger et al., 2017). The neuroprotective properties of this hormone are likely conveyed through anti-apoptotic, anti-inflammatory, antioxidative pathways and through its effects on angiogenesis. The duality between its erythropoietic and non-erythropoietic (or tissue protective) effects likely stems from EPO having two different receptor types (McVicar et al., 2011).

### 2.3 Glucagon-Like Peptide-1

Glucagon-like peptide-1 (GLP-1) is first and foremost recognized as an incretin hormone that regulates glucose-dependent insulin secretion. GLP-1, at the same time, can be detected in the mammalian retina (expression confirmed in the rat retina by Zhang et al., 2009; and in the human retina by Hebsgaard et al., 2018). GLP-1 has been found to convey a wide array of neuroprotective effects in retinal diseases (see Section 5 for more information). While the GLP-1R is undoubtedly present in retinal tissue (Zhang et al., 2009; Zhang et al., 2011; Hebsgaard et al., 2018; see row 4 of Table 1), locating and identifying specific GLP-1R expressing cell types have proven quite challenging as the specificity of the antibodies used to label GLP-1R have been called into question (Hebsgaard et al., 2018). So far, GLP-1R has been localized to the inner retina, mainly to the GCL and to MCs in the rat retina (Zhang et al., 2011) and the GCL in humans (Hebsgaard et al., 2018). GLP-1R agonists, including exenatide, liraglutide, lixisenatide, semaglutide and others, are already routinely used in the treatment of diabetic retinopathy (Pang et al., 2018). The GLP-1 receptor is, to our current understanding, a G-protein coupled receptor that acts through the regulation of adenylate cyclase activity and protein kinase A (PKA), activating the PI3K and mammalian target of rapamycin (mTOR) pathways (Carlessi et al., 2017).

### 2.4 Neuropeptide Y

Neuropeptide Y (or NPY for short) is one of the most well-known on the list of neuroprotective peptides. It has been first isolated by Tatemoto in 1981 from porcine nervous tissue (Tatemoto and Mutt, 1981) and later from the human retina (Straznicky et al., 1992). It belongs to the NPY family of peptides with a series of different NPY receptor subtypes sorted into three different subfamilies (Larhammar et al., 2001). Out of the different subtypes, Y1 and Y2 appear to be the most common in the retina with Y4 and Y5 present in certain non-neuronal cell types (Santos-Carvalho et al., 2014; reviewed in detail by Santos-Carvalho et al., 2015) (see row 5 of Table 1). NPY receptors are thought to act mostly through the inhibition of adenylate cyclase and the cAMP pathway (Martins et al., 2015), although the Y2 and Y4 receptors have been shown to couple to Gq in rabbit smooth muscle cells (Misra et al., 2004). NPY, in general, exerts its effect through the reduction of Ca^2+^ influx and moderates changes in intracellular Ca^2+^ levels (D’Angelo and Brecha, 2004; Alvaro et al., 2009). In the mouse retina, NPY is expressed by both ACs and displaced ACs (dAC), which is very similar to the labelling experienced in rats (Sinclair and Nirenberg, 2001). In the human retina, NPY is also expressed by ACs and dACs (Christiansen et al., 2018a).

### 2.5 Pituitary Adenylyl Cyclase-Activating Polypeptide

Pituitary adenylyl cyclase-activating polypeptide (PACAP) has first been isolated from the hypothalamus in 1989 (Miyata et al., 1989) and come in two forms based on amino acid length, namely PACAP-38 and PACAP-27, with PACAP-38 (referred to simply as PACAP going forward) being the target of extensive research for its neuroprotective capabilities (reviewed by Gábriel et al., 2019). PACAP belongs to the same family as vasoactive intestinal polypeptide (VIP), together with glucagon and secretin. There are three different receptors conveying the effects of PACAP, named PAC1-R (highest affinity), VPAC1-R and VPAC2-R. The signal pathways activated by PACAP receptors are well mapped and PAC1-R and VPAC-1R are known to act both through the activation of adenylate cyclase/PKA/MAPK axis and the phospholipase/inositol trisphosphate/protein kinase C (PLC/IP3/PKC) axis, while VPAC-2R has only been connected to adenylate cyclase activation (Gábriel et al., 2019). In the rat retina, PACAP is expressed by ACs, GCs and HCs while the PAC1-R has been localized to ACs, GCs and the MCs (Izumi et al., 2000; Dénes et al., 2014; see row 6 of Table 1). PACAP is also able to interfere with the stress response through the stimulation of corticotropin releasing hormone secretion (Agarwal et al., 2005). A glucocorticoid analogue, namely dexamethasone, proved its long-term and safe effect in diabetic macular edema treatment. PACAP can impact the hypothalamo-pituitary axis and could counterbalance the effects of corticotropin releasing hormone activity, thus influencing damage caused by diabetic conditions (Bucolo et al., 2018).

VIP is expressed by ACs in the retina (Pérez de Sevilla Müller et al., 2019). Originally, VIP has been described as a vasodilatator but possesses a variety of other functions that it exerts through the VPAC1-R and VPAC2-R (Atlasz et al., 2010; reviewed by Cervia et al., 2019). However, the effectiveness of VIP retinoprotective functions seem to fall short compared to PACAP (Szabadfi et al., 2012a).

### 2.6 Somatostatin

Somatostatin (SST), also called somatotropin release inhibiting factor, is yet another peptide hormone that has been proven to possess neuroprotective properties. SST is expressed in the retina in both of its known active forms (SST-14 and SST-28). SST is produced mainly by the RPE and GABA-ergic ACs (Cristiani et al., 2002; Hernández et al., 2014; Postyeni et al., 2021) and its receptors are present on the PRs, HCs, ACs and RGCs (Cervia et al., 2008a; see row 7 of Table 1). The five G-protein coupled SST receptor types (sst1-5) are all present in the human retina according to the work published by Pérez-Ibave et al. (2019). Li and colleagues suggest that sst5 exerts its effect through the NO/cGMP/protein kinase G pathways and the suppression of T-type Ca^2+^ channels (Li et al., 2019), while sst4 was proposed to modulate L-type Ca^2+^ channels instead (Farrell et al., 2014). Sst2 also acts through the production of cGMP (Mastrodimou et al., 2006) and Thermos and colleagues proposed that sst1 likely acts as an autoreceptor (Thermos et al., 2006).

SST receptor mRNA levels have been specifically measured in the mouse retina, suggesting the abundant expression of sst2 and sst4, accompanied by the less prominent expression of sst1 and very low mRNA levels for sst3 and sst5 overall (Cristiani et al., 2002). Immunoreactivity for sst2A was observed in rodBC, HC and AC, including tyrosine hydroxylase immunoreactive, GABA-erg and glycinerg ACs. SST labeling has been demonstrated in ACs and dACs in the mouse retina but not in GCs, similar to rats (Cristiani et al., 2002).

### 2.7 Substance P

Another important peptide with significant neuroprotective effects is substance P (SP), a member of the tachykinin peptide family. As a neurotransmitter, SP is known to have excitatory effects on both amacrine and retinal ganglion cells (Zalutsky and Miller, 1990). As a neuromodulator, SP stimulates dopamine release (Casini et al., 2004). Out of the three tachykinin receptor types (neurokinin 1, 2 and 3 receptors, abbreviated as NK1, NK2 and NK3, respectively), only NK1 and NK3 are present in the retina. NK1 receptors (with the highest affinity for SP) are found on ON BCs and a diverse population of ACs while NK3 appears to be reserved for ON BCs (Oyamada et al., 1999; Kim et al., 2005; see row 8 of Table 1). Interestingly, there seems to be considerable difference between NK1 expression patterns among different mammalian retina models both during development and in adult animals (Catalani et al., 2006). Neurokinin receptors all belong to the group of G-protein coupled receptors and they are responsible for the activation of PLC and adenylate cyclase, and carry the potential to bind all three neurokinin ligands with different affinity: in addition to SP being the preferential ligand of the NK1 receptor, neurokinin A and B are the preferential ligands of the NK2 and NK3 receptors, respectively. MAPKs, ERK1/2 and mTOR and NF-κB are all involved in SP-related signaling through the NK1 (Mashaghi et al., 2016).

In the mouse retina, SP expression has been localized to ACs (Haverkamp and Wässle, 2000). NK1 receptors are expressed by ON bipolar cells (Catalani et al., 2004), although the same labelling isn’t present in rats (Casini et al., 1997; Casini et al., 2000). However, the NK1 expression patterns in ACs appear similar in both species and they are present in a diverse group of ACs, including co-localization with GABA, tyrosine hydroxylase and SP (Casini et al., 2000; Catalani et al., 2004). In humans, SP immunoreactivity was also detected in dACs and GCs (Cuenca et al., 1995).

### 2.8 Additional Factors

In addition to NPs, a variety of other molecules contribute to the development and maintenance of a healthy retina. Similar to the peptides listed above, the mammalian retina expresses a number of growth factors, hormones and other neurotrophic proteinaceous substances. The brain-derived neurotrophic factor (BDNF) is present in the retina and contributes to both retinal development and health. BDNF is produced by a variety of neurons (PRs, RGCs, ACs) and glia (MCs) alike (Bennett et al., 1999; Oku et al., 2002; Vecino et al., 2002; Avwenagha et al., 2006) and it exerts its effects through a tyrosine kinase receptor, tropomyosin receptor kinase B (TrkB) (reviewed by Fudalej et al., 2021). TrkB has the highest affinity for BDNF as its ligand among growth factors and affects MAPK activity and the PI3K and the PLC gamma pathways (reviewed by Huang and Reichardt, 2003). In the retina, the TrkB receptor is expressed by PR, AC and RGCs (Cellerino and Kohler, 1997; Vecino et al., 1998; Vecino et al., 2002; see row 9 of Table 1). Pigment epithelium derived factor (PEDF) is produced by the cells of the RPE, with its receptor (PEDF-R) simultaneously present on cells of the RPE and PRs (see row 10 in Table 1). As a consequence, PEDF exerts its protective effects mainly on PRs. Our current knowledge on PEDF action has been reviewed by Rebustini et al. (2021). So far, several potential targets have been reported for the pathways involved in PEDF signaling, including MAPK activation, JAK/STAT, PI3K and additional pathways (Pagan-Mercado and Becerra, 2019). Vascular endothelial growth factors (VEGF-A, B, C and D) are molecules with proangiogenic effects. VEGF can be produced by multiple cells of the retina, including MCs, astrocytes, RGCs and the epithelial cells of the retinal vasculature and the RPE (Saint-Geniez et al., 2008; see row 11 in Table 1). While VEGF seems to convey a certain amount of neuroprotection on PRs (Suzuki et al., 2011) and RGCs (Froger et al., 2020), it is also responsible for facilitating pathological neovascularization in the retina. VEGFs exert their effects through tyrosine-kinase type receptors (VEGF-R). VEGF-R1, VEGF-R2 and VEGF-R3 are all expressed in the retina, although VEGF-R2 and R3 expression appears to be upregulated in pathological conditions (Witmer et al., 2002). It is important to note that all VEGF receptors all possess tyrosine kinase activity. VEGF-R2 is considered to be the most important mediator of VEGF-related angiogenesis. After autophosphorylation, the MAPK pathway is activated through PLC gamma (Takahashi et al., 2001). Receptor activation also affects the PI3K pathway, among others (Kowanetz and Ferrara, 2006).

## 3 Genetically Modified Experimental Models in the Study of Retinal Health

In the last few decades, mouse models played a prominent role in furthering our understanding of the cause and effect of retinal diseases as they share several similar biochemical, physiological and anatomical features with the human retina. Now that the methodology for genetic manipulation is already well-established, it has become an immensely powerful tool that provides detailed step-by-step analysis of disease progression and pathophysiology. At the moment, the MouseMine database contains more than 230 different mouse models connected to different retinal diseases. In fact, the very targets of these investigations could be the individual retinal cell types and retinal synaptic connections. For that, the manipulation of different mouse models is ideally based on a construct with suitable cell-specific promoters, where the transgene is expressed under strict control or after specific induction (e.g., tissue-specific drug-induction). As an option, we can create knockout (KO) models (conditional or conventional), where the inactivation of the target gene helps us understand the biological function of the now missing gene product. As another option, random exogenous transgene integrations, gene knock-in or target gene overexpression are also useful approaches for studying the actual changes of different biological traits. In the perspective of complex metabolic retinal pathologies, the use of these methods cannot only be based on the well-known genetic background of diseases but, with these technologies, it is certainly achievable to describe or modify the specific pathways and mediators involved. Genetically modified mouse lines offer new insight in understanding the altered behavior of different retinal cell types and the changing expression of signal molecules in the manifestation of retinal impairments. The protective effect of NPs could also be made traceable in different disease models at multiple molecular levels. Although there are many studies about the promising effect of NPs and the usefulness of the transgenic approaches in retinal diseases, their combination in the research process remains limited. In the present work, we try to collect available knowledge on the different genetic models in metabolic retinal diseases (DR, ischemia) listing advantages and supplementing with promising therapeutic effects of different NPs.

### 3.1 Genetic Models in the Research of Neuropeptide Function in the Retina

The past decades have seen an increase in the use of genetically modified animal models in retinal research, which improved our knowledge of the cell-specific developmental, degenerative and rescue processes. They have been widely used in investigations of PR development (Fong et al., 2005) and degeneration (Geiger et al., 1994; Joseph and Li, 1996; Kanan et al., 2010; Tolmachova et al., 2010; Lipinski et al., 2011; Wu et al., 2012; Roman et al., 2018; Zhong et al., 2020); BC (He et al., 2019; Yang et al., 2019), MC (Peant et al., 2007) and GC degeneration (Husain et al., 2014; Wang L. et al., 2020) and also in rescue processes (McNally et al., 1999; Dong et al., 2007; Liu et al., 2012; Garcia-Caballero et al., 2018; Wang et al., 2019). However, the detailed discussion of genetic disorders leading to retinal degeneration are beyond the scope of the present review. At the same time, alterations in NP expression could also be achievable through these genetic models in the retina, offering additional information on normal retinal physiology, as well as the therapeutic role of NPs in pathologies. Indeed, changes in peptide homeostasis elicited by genetic means clearly causes alterations in retinal microanatomy or physiology. For example, overexpression of PAC1R caused a decline in GABAergic AC production (Lang et al., 2010), while overexpression of BDNF modified RGC dendritic branching (Liu et al., 2007). Deletion of the PAC1 receptor has caused a deficit in RGC numbers (Van et al., 2021), and BDNF+/− mice also showed lower cell numbers in the ganglion cell layer and a decline in the inner retinal function observable in the form of decreased amplitude of positive scotopic threshold responses (Gupta et al., 2014). Research carried out in NP gene KO models has demonstrated the prominent role of PACAP in retinal age-related changes (Kovacs-Valasek et al., 2017). A BDNF KO model has been used to emphasize the role of BDNF in RGC development (Cellerino et al., 2003). The physiological function and distribution of VIP in retinal cells has also been investigated by transgenic technology (Park et al., 2015; Pérez de Sevilla Müller et al., 2019), as well as the role of orexins in retinal function via influencing dopaminergic cells (Qiao et al., 2017). Interaction between peptides can also be turned into a powerful tool; in the BDNF KO model, the BDNF-mediated VIP expression has been studied in ACs (Ladewig et al., 2004). Repeated intravitreal injections of PACAP caused elevated somatostatin containing cell numbers in SST transgenic mice in the aging retina (Postyeni et al., 2021). To reveal the specific effect of neuropeptides exerted in retinal pathologies, the design of new models was mandated, which has also helped recognize the complex biological importance of NPs.

## 4 Metabolic Retinal Diseases: Pathology and Neuropeptides

Normal retinal function is essential for maintaining visual perception, meaning that any disease or injury that threatens retinal integrity is a cause for substantial concern. Diseases of metabolic origin pose an especially serious problem for people worldwide (Rodríguez et al., 2021; Simó et al., 2021) and the need to find treatment for these conditions is greater than ever. NPs have also been utilized in the fight for maintaining retinal integrity—with varying efficiency (see Table 2).

**TABLE 2:** Effect of neuropeptides on retinas affected by diabetic retinopathy and ischemia.

Effect of neuropeptides on retinas affected by diabetic retinopathy and ischemia									
Row	Peptide	Effect on retinal pathophysiology^a^						Effect on disease progression	
1		Apoptosis or cell death	Oxidative stress	Autophagy	Immune response and inflammation	BRB breakdown	Neovasularization (VEGF expression)	Diabetic retinopathy	Ischemia
2	AngII		Pro (Wilkinson-Berka et al., 2013)		Pro (Wilkinson-Berka et al., 2019)		Pro (Otani et al., 2000)	Progression (Choudhary et al., 2016)	Progression (Fukuda et al., 2010)
3	Ang (1-7)	Anti (Verma et al., 2012)^b^	Anti (Verma et al., 2012)^b^		Anti (Verma et al., 2012)^b^			Likely protective (Verma, 2012)^b^	
4	EPO	Anti (Luo et al., 2015; Reid and Lois, 2017)	Anti (Luo et al., 2015)		Anti (Reid and Lois, 2017; Xie et al., 2021)	Anti (Xu et al., 2014)	Conflicting results (Reid and Lois, 2017) anti (Bretz et al., 2020)^b^	Conflicting results	Protective (Junk et al., 2002; Mowat et al., 2012)
5	GLP-1	Anti (Zhang et al., 2011; Fan et al., 2014a; Hernández et al., 2016)	Anti (Ramos et al., 2020^b^; Zhou et al., 2021)	Inhibits (Cai et al., 2017)	Anti (Gonçalves et al., 2016; Chung et al., 2020)	Anti (Fan et al., 2014b; Gonçalves et al., 2016)	Anti (Fan et al., 2014b)	Conflicting results (Marso et al., 2016; Simó and Hernández, 2017; Bain et al., 2019; Saw et al., 2019; Dauner and Farley, 2021)	Protective (Gonçalves et al., 2016)
6	NPY				Anti (Ferreira et al., 2010)		Conflicting results, anti in DR (Ou et al., 2020)	Likely protective (Ou et al., 2020)	Conflicting results (Christiansen et al., 2018)
7	PACAP	Anti (Atlasz et al., 2010; Szabadfi et al., 2014; Szabadfi et al., 2016)			Anti (Werling et al., 2016; D’Amico et al., 2017)	Anti (Maugeri et al., 2019)	Anti in DR (D’Amico et al., 2017; Maugeri et al., 2017) no effect in ischemia (Schmid et al., 2012)^b^	Protective (Szabadfi et al., 2016; Gábriel et al., 2019; Maugeri et al., 2019)	Protective (Atlasz et al., 2010; Danyadi et al., 2014; Werling et al., 2014; Vaczy et al., 2016; Werling et al., 2016; Werling et al., 2017)
8	SST/OCT	Anti (Dal Monte et al., 2012^b^; Wang et al., 2015; Amato et al., 2016)	Anti (Wang et al., 2015)	Inhibits (Amato et al., 2018)	(Hernandez et al., 2020)^b^		Anti (Cervia et al., 2012^b^; Amato et al., 2020)	Protective (Amato et al., 2018)	Protective (Mastrodimou et al., 2005; Wang et al., 2017)
9	SP	Anti (Yang et al., 2013)	Anti (D’Alessandro et al., 2014)		Anti (Baek et al., 2020)		No effect (Schmid et al., 2012)^b^	Protective (Yang et al., 2013)	Protective (Sakamoto et al., 2017)

Diabetic retinopathy and ischemia can both exert their deleterious effects through multiple pathways, including oxidative stress, inflammation, apoptosis and uncontrolled neovascularization that eventually culminate in the disruption and the destruction of the neurovascular unit. Neuropeptides have been shown to exert protective or destructive capabilities under these pathological conditions.

a For this review, we only considered results obtained from models of DR or ischemia.

b Results obtained from genetically modified animal models.

While the neural elements of the retina are especially sensitive to insult and injury, it is important to remember that functioning glial cells, a steady supply of blood and a healthy blood-retina-barrier (BRB) are all necessary for this organ to function. In many cases, the main reason for the loss or impairment of vision lies in the degradation of the neurovascular unit (the functional connective of neuron, glia and the blood vessels) and the loss of integrity in the blood-retina-barrier, which leads to the perturbation of the retinal microenvironment (Simó et al., 2021). Diseases of metabolic origin tend to attack on multiple fronts, making it even harder to combat their effects; the so-called deleterious effects often manifest themselves through the production of reactive oxygen species (ROS), taking substantial resources from the body to combat oxidative stress (Kowluru and Chan, 2007). Inflammation (and the activation of the immune system) is also a common factor in disease pathology (Whitcup et al., 2013). Eventually, the accumulated damage leads to either necrosis or apoptosis. Any damage to the neurovascular unit will threaten retinal integrity as a whole, so pathological processes affecting retinal vasculature or the BRB are extremely dangerous (Erickson et al., 2007; Nian et al., 2021). Because of the high metabolic demand, the retina has extensive capabilities for neovascularization that can quickly spiral out of control when growth factor (mainly VEGF) expression runs unchecked, which provides yet another vulnerability for diseases to exploit (Campochiaro, 2013). Over the course of time and in small, manageable quantities, many of these aspects also contribute to the natural process of aging or senescence (see Figure 10 in Kovács-Valasek et al., 2021).

### 4.1 Diabetic Retinopathy

Diabetic retinopathy (DR) results from the extensive damage caused to the retinal vasculature and tissue by diabetes mellitus (DM). Because of the high prevalence of DM in modern society, DR has been identified as a major health concern and one of the leading causes of blindness worldwide (Simó-Servat et al., 2019). In patients with diabetes, the increased glucose influx causes an overproduction of advanced glycation end-products (Milne and Brownstein, 2013) and ROS (Sasaki et al., 2010; Kang and Yang, 2020). In the early (or non-proliferative) stage, the pericytes are the first to fall (Romeo et al., 2002), only to be followed by the epithelial cells soon after, leading to a dangerous increase in BRB permeability (Beltramo and Porta, 2013). The degeneration and death of the neuronal elements, however, starts separately and not as a direct result of the damage to the retinal vasculature (Wang and Lo, 2018). In the meantime, glial activity is altered and the micro and macroglia of the retina participate in the development of the immune response (Altmann and Schmidt, 2018). In the late or proliferative stage, the release of growth factors (VEGF) causes uncontrolled neovascularization (Tomita et al., 2021). One of the pathways involved in VEGF-related signaling in pericytes seems to be connected to prostaglandine-endoperoxidase synthase activity, which likely exerts feed forward regulation on VEGF secretion, further aggravating the damage (Giurdanella et al., 2015). As a consequence, DR is characterized by the appearance of microaneurysms and hemorrhages in the retina, the apoptosis of pericytes and endothelial cells, and neovascularization.

Regarding the possible role of neuropeptides, studies on one hand confirmed increased expression for the components of the ACE/AngII/AT1R axis in the diabetic retina (reviewed by Fletcher et al., 2010; further supported by research carried out in mice and rats by Verma et al., 2012). To assess the potential of angiotensin receptor (ATR) blockers in the treatment of DR, a clinical trial using candesartan has been carried out (DIRECT Prevent and Protect trials, Chaturvedi et al., 2008; Sjølie et al., 2008). While the ATR blockade through candesartan could only elicit DR prevention in type 1 and disease regression in type 2 diabetes (most effective in the early stages of DR), the development of microaneurysms was hindered in both diabetes type 1 and 2 (Sjølie et al., 2011). In the Renin-Angiotensin System Study, both ATR blocking effects (losartan) and ACE inhibition (enalapril) were studied in type 1 diabetes. The progression of DR was reduced in both cases (Mauer et al., 2009; Harindhanavudhi et al., 2011). While DIRECT and RASS presented conflicting results in certain areas (Harindhanavudhi et al., 2011), the conclusion seems to be that the ACE/AngII/AT1R axis does play a small but non-negligible role in the development of DR. An enhanced expression of ACE2/Ang (1-7) was shown to be beneficial in alleviating the effects of DR in animal models (Verma et al., 2012). While the therapeutic potential of Ang (1-7) seems promising considering its protective effects (anti-apoptotic, anti-oxidative, anti-inflammatory; see Table 2), its short half-life makes it hard to utilize (Verma et al., 2020).

Similarly, patients affected by DR express EPO and its receptors in higher quantities compared to healthy human donors (Hernández et al., 2006; Shah et al., 2009). The usability of EPO as a therapeutic agent also proposes some controversies; single nucleotide polymorphisms in the EPO gene have been examined, yielding different results on whether different alleles present increased, decreased or caused no susceptibility to DR (reviewed by Reid and Lois, 2017). It is still up to debate whether the high vitreal concentration of EPO is an agent of disease pathology or a compensatory mechanism activated to combat the deleterious influence of DR. On the other hand, the expression of the majority of neuroprotective peptides appear to be downregulated in diabetic retina, including GLP-1 (reviewed by Pang et al., 2018), NPY (studied in human and mice cell cultures and rat retinal explant cultures by Ou et al., 2020), PACAP (research carried out in rats by Giunta et al., 2012), SST (studied in human diabetic retinas by Carrasco et al., 2007). Verma and colleagues have shown in mouse and rat models that during the early stages of diabetes, the vasoprotective components of the retinal RAS are expressed at higher levels but fail to compensate for the progression of the disease and are eventually downregulated (Verma et al., 2012). SP levels were also found to decrease under diabetic conditions and the restoration of endogenous SP helped prevent apoptosis in streptozotocin-induced (STZ) model of diabetes in rats (Yang et al., 2013). This alludes to the possibility that the damage caused by DR might, at least in part, stem from the underproduction of neuropeptides in the retina.

#### 4.1.1 Effect of Neuropeptides on Disease Progression

In this section we examine the role of neuropeptides on oxidative stress, the immune response and vascular alterations caused in the diabetic retina. Recently conducted research provides evidence that GLP-1 possesses anti-oxidant capabilities against oxidative stress in the diabetic retina and it is capable of affecting DNA repair and neurogenesis to a certain level in mice (Ramos et al., 2020). With regards to GLP-1RA, exanatide-4 (E4) and other analogs were able to reduce apoptotic damage and ameliorate the decrease in cell numbers in diabetic rat retinas (Zhang et al., 2011; Fan et al., 2014a). Liraglutide, in addition, was also able to alleviate apoptotic damage to some degree in the human and mouse retina (Hernández et al., 2016). According to Zhou and colleagues, the beneficial effects of liraglutide also manifest in the mitigation of mitochondrial damage, supposedly through the PTEN-induced kinase/Parkin pathway (research carried out in rats by Zhou et al., 2021). Studies validated the protective effects of EPO against cell loss among pericytes and neurons of the retina (Wang et al., 2011; research carried out in rats by Zhang et al., 2009). PACAP, one of the most versatile agents, upregulates anti-apoptotic and downregulates pro-apoptotic pathways in DM and seems to have an autoregulating effect on PAC1R expression in rats (Szabadfi et al., 2014; Szabadfi et al., 2016). PACAP also exerts protection over the neuronal elements in the GCL and dopaminergic ACs of the rat retina (Atlasz et al., 2010; Szabadfi et al., 2012b).

While experimental work on the protective effects of SST provided favorable results, because of the BRB, however, SST or SST analogs delivered through the bloodstream could not be used for a therapeutic approach. On one hand, topical administration of SST was successful in conveying neuroprotective effects in rats (Hernández et al., 2013). On the other hand, octreotide (OCT), probably the most commonly used SST analog, offers promising prospects in the treatment of DR. Since treatment through intravitreal injections carries certain risks, OCT has also been utilized in the research of intraocular nanoparticle delivery (Amato et al., 2018; Amato et al., 2020). OCT has been demonstrated to reduce apoptosis as well in mouse retinal explants (Amato et al., 2016) and SP injections were capable of restoring and increasing retinal nerve fibre layer thickness in diabetic rats while suppressing apoptosis (Baek et al., 2020).

Immune response seems to be crucial in symptom development. So far, a reduction in ACE2 has been demonstrated to reduce the inflammatory response in cell cultures of the RPE (Fu et al., 2017). In addition, studies connected EPO to reduced inflammatory cytokine production in cell cultures and rats (Lei et al., 2011; McVicar et al., 2011). Some of its effects are likely mediated through the inhibition of microglia activation through the Src/Akt/cofilin pathway according to research carried out in rats (Xie et al., 2021). EPO might also be able to protect rat MCs through the BDNF/TrkB pathway during hyperglycemia (Wang and Xia, 2015). In the case of lixisenatide, the known anti-inflammatory effects of GLP-1RA have also been validated in the mouse retina (Chung et al., 2020). Controversy also arose regarding the effects of GLP-1RA when semaglutide was connected to a higher prevalence of DR in patients receiving treatment (SUSTAIN-6 trial, Marso et al., 2016; Simó and Hernández, 2017). Additional studies have resulted in diverging results between GLP-1RAs and DR prevalence (Dauner and Farley, 2021), with an ongoing discourse on whether the increased prevalence can be explained by the sudden drop of glucose levels at the initiation of treatment (Bain et al., 2019). So far, multiple reviews encompassing the GLP-1RA effect on DR have been published (Pang et al., 2018; Saw et al., 2019). Furthermore, according to our current knowledge, NPY has the capability to suppress the production of NO and the pro-inflammatory cytokine interleukin 1 beta (IL-1β) in microglia through the Y1 receptor (research carried out in cultured cells by Ferreira et al., 2010). Similarly, PACAP has also demonstrated capacity for decreasing IL-1β expression in rats (D’Amico et al., 2017). Additionally, PACAP has been able to downregulate hypoxia-inducible factor expression in diabetic rats (D’Amico et al., 2015). The beneficial effects of SP against the deleterious effects of DR seem to be the consequence of immunosuppression as well (Baek et al., 2020).

Partially due to the increased immune response, BRB breakdown is initiated and as a consequence, vascular permeability is increased in DR. Additionally, this process is also connected to increased VEGF expression. Among the peptides, Ang II has been capable of increasing VEGF expression (Otani et al., 2000) and the migration of bovine pericytes (Nadal et al., 1999) when signaling through the AT1R. Regarding EPO, a number of studies have showed an inhibitory effect of externally administered EPO on VEGF expression in rats (Wang et al., 2011; Mitsuhashi et al., 2013) and it has also been helpful in preventing BRB breakdown in a comparative study (Xu et al., 2014). E4 also acts toward maintaining BRB integrity, downregulates VEGF expression in rats (Fan et al., 2014b) and mitigates the harmful effects of advanced glycation end in RPE cells (Dorecka et al., 2013). These effects are at least partially conveyed through sirtuin pathways as E4 treatment has caused an increase in sirtuin (SIRT1 and SIRT3) expression in rats (Zeng et al., 2016). In the meantime, Al Sabaani has demonstrated that E4 likely exerts its protective effects on RPE cells under hyperglycemia through the suppression of P66Shc and the inhibition of c-Jun N-terminal kinase and PKC beta activity (Al Sabaani, 2021). NPY has been demonstrated to counteract VEGF functions and protect vascular integrity through the inhibition of the MAPK pathway (Ou et al., 2020). PACAP is partially able to elicit protective effects against DR in the RPE through the activation of the epidermal growth factor receptor (Maugeri et al., 2019). PACAP has also been found to inhibit hyperglycemia-induced endothelial cell proliferation in cell cultures (Castorina et al., 2010), and VEGF expression in rats and cell cultures (D’Amico et al., 2017; Maugeri et al., 2017). Last but not least, OCT has been demonstrated to reduce VEGF expression in an *ex vivo* mouse model of DR (Amato et al., 2016). In the early stages of DR, the protective effects of OCT seem to be in part conveyed through the moderation of autophagy (Amato et al., 2018). Similarly, GLP-1 can also inhibit autophagy (Cai et al., 2017).

#### 4.1.2 Animal Models in Diabetic Retinopathy

The current treatment of DR is based on different approaches (vid. anti-VEGF injections, laser treatments, surgery) and recently, several studies have demonstrated the potential curative potential of different neuropeptides in DR (see above). Hidden in the background of the functional and structural changes of the retina, there is a strong connection between vascular impairments (vasoregression, BRB breakdown, altered hemodynamics) and retinal neurodegeneration (neuron apoptosis, glial dysfunction) (Simó et al., 2014; Rossino et al., 2019), the two main characteristic features of the pathophysiology of DR. Several studies have described that neurodegeneration in the retina already occurs before the vascular changes take place, which makes research aimed at new, promising neuroprotective factors markedly important (Abcouwer and Gardner, 2014; Simó et al., 2014; Simó and Hernández, 2015). Perhaps this is why it has been suggested that gene therapies could target neuroprotection and vasculopathy (Wang J. H. et al., 2020).

Both induced diabetes (e.g., streptozotocin or high-sugar diet induced) and different strain-specific models (e.g., *db/db*, *Ins2* ^*Akita*^, *Pdgrfr* ^*redeye*^) with well characterized mutations in one well known gene (e.g., leptin receptor gene, insulin gene, platelet derived growth factor gene), or transgenic models offer excellent research opportunities. In previous works, different neuropeptides have shown promising curative effects in all of the above-mentioned consequences of DR in the retina (Wilkinson-Berka et al., 2019). Genetic models have made it possible to target the molecular processes of these retinal alterations or explore promising therapeutic pathways from different angles; they can highlight new causative events and/or prospective mediators in processes of inflammation (Bogdanov et al., 2017; Sigurdardottir et al., 2019; Zapadka et al., 2020; Chen et al., 2021; Crespo-Garcia et al., 2021), vascular alterations (Kern et al., 2010; Zhou et al., 2017; Zhu et al., 2018; Lindstrom et al., 2019; Sigurdardottir et al., 2019; Ivanova et al., 2020; Tecilazich et al., 2020), oxidative stress (Kowluru et al., 2006; Bogdanov et al., 2017; Dierschke et al., 2019; Shao et al., 2019; Sigurdardottir et al., 2019; Chen et al., 2021), VEGF production (Lin et al., 2011; Fu et al., 2015; Yin et al., 2021) and the formation of advanced glycation end products (or their receptor) and neuronal/glial cell degeneration (Kezic et al., 2013; Yang et al., 2015; Chen et al., 2021). The contribution of pro-angiogenic VEGF isoforms to the pathomechanism of DR has also been explored using Ins2^Akita^ mice (Bucolo et al., 2021).

#### 4.1.3 Neuropeptides in Genetically Modified Models of Diabetic Retinopathy

BDNF has been found to be a promising agent against DR; intravitreal injections of BDNF rescued dopaminergic ACs in induced diabetes (Seki et al., 2004; Wan et al., 2010). Interestingly, both BDNF production and TrkB receptor expression have been observed to decrease in the diabetic retina (Seki et al., 2004; Ola et al., 2013), similar to the decrease in protective NPs in DR. In VEGF2 receptor KO mice, a reduction occurred in retinal BDNF production, leading to MC loss (Fu et al., 2015). Additionally, the knockdown of TrkB has been shown to cause the downregulation of survival mediators (e.g., protein kinase B, extracellular signal-regulated kinases; Le et al., 2021). In a different set of experiments, increased expression of ACE2/Ang-(1-7) in endothelial nitric oxide synthase^−/−^ (*eNOS* ^*−/−*^) mice with STZ-induced diabetes protected against complications of DR (e.g., infiltrating inflammatory cells, vascular leakage, oxidative damage; Verma et al., 2012). Underproduction of SST during diabetes has also been described (Carrasco et al., 2007), and treatment with SST prevented inflammation, glial activation, and neurodegenerative and functional alterations in *db/db* mice retinas (Hernández et al., 2020). In *db/db* mice, the topical administration of GLP-1 has reduced oxidative stress and promoted the repair of DNA damage (Ramos et al., 2020). Furthermore, treatment with the GLP-1RA liraglutide has prevented the upregulation of proapoptotic/proinflammatory signals (Hernández et al., 2016). In light of the above section, it should be noted that the application of true transgenic methods in the research of NP effect on DR is still infrequent, although the methodology is available and has already proven its usefulness.

### 4.2 Ischemia

Ischemia refers to a condition where restricted blood supply leads to tissue and cell damage. Ischemic damage results both from decreased oxygen levels and a shortage in nutrients necessary for cell survival, in addition to the accumulation of toxic metabolites (Osborne et al., 2004). To make things worse, restoring normal blood flow after ischemia results in even more damage, referred to as the ischemia reperfusion injury (IRI) which results from the combined deleterious effects of oxidative stress and inflammation. Glutamate excitotoxicity is a long-term effect of ischemia and recognized as a major contributor to ischemic retinal cell death; simultaneously, hypoxia-triggered pathological neovascularization also poses a serious concern (Osborne et al., 2004). Currently utilized treatments specifically aimed to minimize ischemic damage include laser photocoagulation, corticosteroid treatment as a form of immune response suppression, vitreoretinal surgery and intraocular injections of VEGF antibodies (O’Leary et al., 2019).

#### 4.2.1 Effect of Neuropeptides on Disease Progression

Considering how ischemia can be used as a model for various pathological processes, the possible protective role of NPs have also been researched in multiple animal models. Just like in DR, certain peptides appear to have quantifiable effects in various aspects of neuroprotection, including anti-oxidant and anti-inflammatory capabilities and the potential to ameliorate the disrupting effects of IRI. For example, OCT has been demonstrated to exert protective effects in the mouse retina against oxidative stress, which at least partially stem from mitigating oxidative damage through decreasing NF-κB p-p65 and intercellular adhesion molecule 1 (ICAM-1) levels (Wang et al., 2015). SP treatment in ischemic retinas was also demonstrated to upregulate antioxidant levels (D’Alessandro et al., 2014). Furthermore, OCT is also known to prevent apoptosis following ischemia (Wang et al., 2015). Additionally, E4, a GLP-1RA of high importance, has been able to exert anti-inflammatory effects in the ischemic rat retina (Gonçalves et al., 2016). E4 also appears to protect against BRB breakdown (Gonçalves et al., 2016) and it has been shown capability to regulate capillary diameter through vasodilation. While some experimental work supports the NO-mediated dilatator effect of E4 through the PI3K/eNOS/NO-cGMP pathway (research conducted in rats by Zhai et al., 2020), other studies do not (study conducted on human patients by Smits et al., 2015).

As ischemia is also known to affect the retinal vasculature, candesartan, one of the important ARBs, has been studied for its potential effect of mitigating ischemic damage through their effect on angiogenesis. Interestingly, conflicting results have been published, with a number of studies reporting pro-angiogenic effects and a supporting effect on VEGF expression, while other groups found these substances to maintain preventive effects against pathological neovascularization (reviewed by Willis et al., 2011; research conducted in mice by Shanab et al., 2015). The work published by Shanab and colleagues seems to resolve the controversy as they found that candesartan promotes reparative angiogenesis but not pathological neovascularization, and the reason for that is that it inhibits inducible nitric oxide synthase expression (Shanab et al., 2015). However, ARBs have proven to be unable to completely negate ischemic damage according to experimental work carried out in rats (Fukuda et al., 2010). Aliskiren, a direct renin inhibitor, was also tested for its effect on ischemia and was found to be more effective in mitigating IRI in the rat retina through the suppression of retinal RAS (Tenkumo et al., 2014).

Furthermore, Christiansen and colleagues tested the effects of intravitreal NPY treatment in a porcine model of acute retinal ischemia and found that it only enhanced ischemic damage (Christiansen et al., 2018b). These observations are markedly important as they contradict earlier results where NPY treatment has been found to convey neuroprotective effects or at least significant change in retinal health could not be observed following treatment (results from cell cultures by Alvaro et al., 2008; earlier experimental work reviewed by Santos-Carvalho et al., 2014; and a study conducted on rat retinas by Martins et al., 2015). Christiansen and colleagues argue that the damaging effect could have been masked in the *ex vivo* models used in earlier studies as NPY has been demonstrated to cause vasoconstriction in the retinal arteries (Prieto et al., 1995; Christiansen et al., 2018b). Regardless, NPY was shown to play no role in pathological retinal neovascularization in the OIR mouse model (Schmid et al., 2012). In the same model, Schmid and colleagues also found that SP was not involved in the pathogenesis of retinal neovascularization despite having angiogenic properties in other tissues (Schmid et al., 2012).

Contrary to the results regarding the lack of NPY and SP participation, another peptide, PACAP has been found helpful in rescuing cells following hypoperfusion, in addition to conveying protective effects in the retina against excitotoxicity (PACAP retinoprotective effects reviewed by Atlasz et al., 2010). Its protective effects under ischemic conditions are further supported by additional and follow-up studies (research carried out in rats by Danyadi et al., 2014; Werling et al., 2014; Vaczy et al., 2016; Werling et al., 2016; Werling et al., 2017). In bilateral common carotied artery occlusion induced ischemia, PACAP was also shown to mitigate damage through the ERK1/2 and Akt pathways, a decrease in inflammatory cytokine production and by acting against MC activation (Werling et al., 2016). Another NP with considerable protective abilities seems to be SST. Although SST itself remains hard to utilize for experimental purposes in ischemia with the BRB intact (study carried out in rats by Mastrodimou et al., 2005), SSTAs and cortistatin were able to induce protective effects under chemically induced ischemia. Wang and colleagues have also explored the protective effects of endogenous SST release triggered by capsaicin in mice (Wang et al., 2017). Capsaicin induced NP release has been reported to protect against NMDA-induced neuronal cell death in the rat retina (Sakamoto et al., 2014), possibly by signaling through NK1 receptors (Sakamoto et al., 2017). This suggests capsaicin activated SP release, similar to the mechanism described for SST by Wang et al. (2017). The possible role of capsaicin as a potent activator of NP release may deserve further attention. Last but not least, it is worth mentioning that ARA290 (an EPO mimetic also referred to as cibenitide) has been reported to possess beneficial effects that could be utilized in the treatment of ischemic retinopathy in cell cultures (O’Leary et al., 2019). We know that ischemia results in increased levels of EPO-R expression in rats (Junk et al., 2002) and elevated concentrations of EPO in the vitreous fluid in mice (Mowat et al., 2012). EPO has been shown to have protective effects against retinal ischemic damage (Junk et al., 2002; Mowat et al., 2012) but the mechanism of its protective actions are not fully understood.

#### 4.2.2 Animal Models in Ischemia

Animal models of induced and spontaneously occurring ischemia are currently in use to describe pathological changes specific to these conditions, and for testing new therapeutic strategies in the retina. These models are built on cerebral artery occlusion (CAO), chronic carotid ligation, the photocoagulation of retinal vessels, endothelin administration, increased intraocular pressure (IOP) and central retinal artery occlusion (Minhas et al., 2012). These models demonstrate how different pathways and mediators of the molecular cascade contribute to the manifestation of ischemia, with the main participants of the pathophysiology originating from the disturbance of the nutrient and oxygen supply. Different genetic approaches used here target vascular alterations (Murinello et al., 2019; Bats et al., 2020; Gutsaeva et al., 2020; Villacampa et al., 2020), oxidative stress mediators (Chan et al., 2012; Schultz et al., 2016), neuronal cell death (Cervia et al., 2008b; Zhu et al., 2013; Luo et al., 2021) and inflammation (Portillo et al., 2008; Dvoriantchikova et al., 2014).

#### 4.2.3 Neuropeptides in Genetically Modified Models of Ischemia

The protective role of different neuropeptides has also been investigated in various pathomechanisms and curative treatments. In the OIR mouse model, PEDF has been observed to prevent β-catenin phosphorylation and the activation of several inflammatory and angiogenic factors. Furthermore, PEDF overexpression has also been shown to attenuate VEGF and β-catenin expression (Park et al., 2011). Moreover, EPO signaling has reduced angiogenesis and vascularization following OIR in mice (Bretz et al., 2020). The expression of NPY and substance P has been observed to decrease in the OIR mouse and they do not appear to play any role in neovascularization, which was further validated in the *NPY KO* mice, where it was found that NPY does, in fact, not take part in vascularization (Schmid et al., 2012). In the *sst1 KO* model, SST and its receptor have played a protective role against hypoxia induced retinal injury by keeping neovascularization under control (Cervia et al., 2012). Moreover, OCT has attenuated electroretinogram alterations and apoptotic signals during hypoxia in the retina of *sst1 KO* mice (Dal Monte et al., 2012). After bilateral carotid artery occlusion, increased damage has been described in PACAP deficient mice compared to wild type mice (Szabadfi et al., 2012c). It is worth noting that at this point in time, there seems to be no true transgenic model available for research aimed at ischemia and IRI.

## 5 Conclusion

**FIGURE 1:**
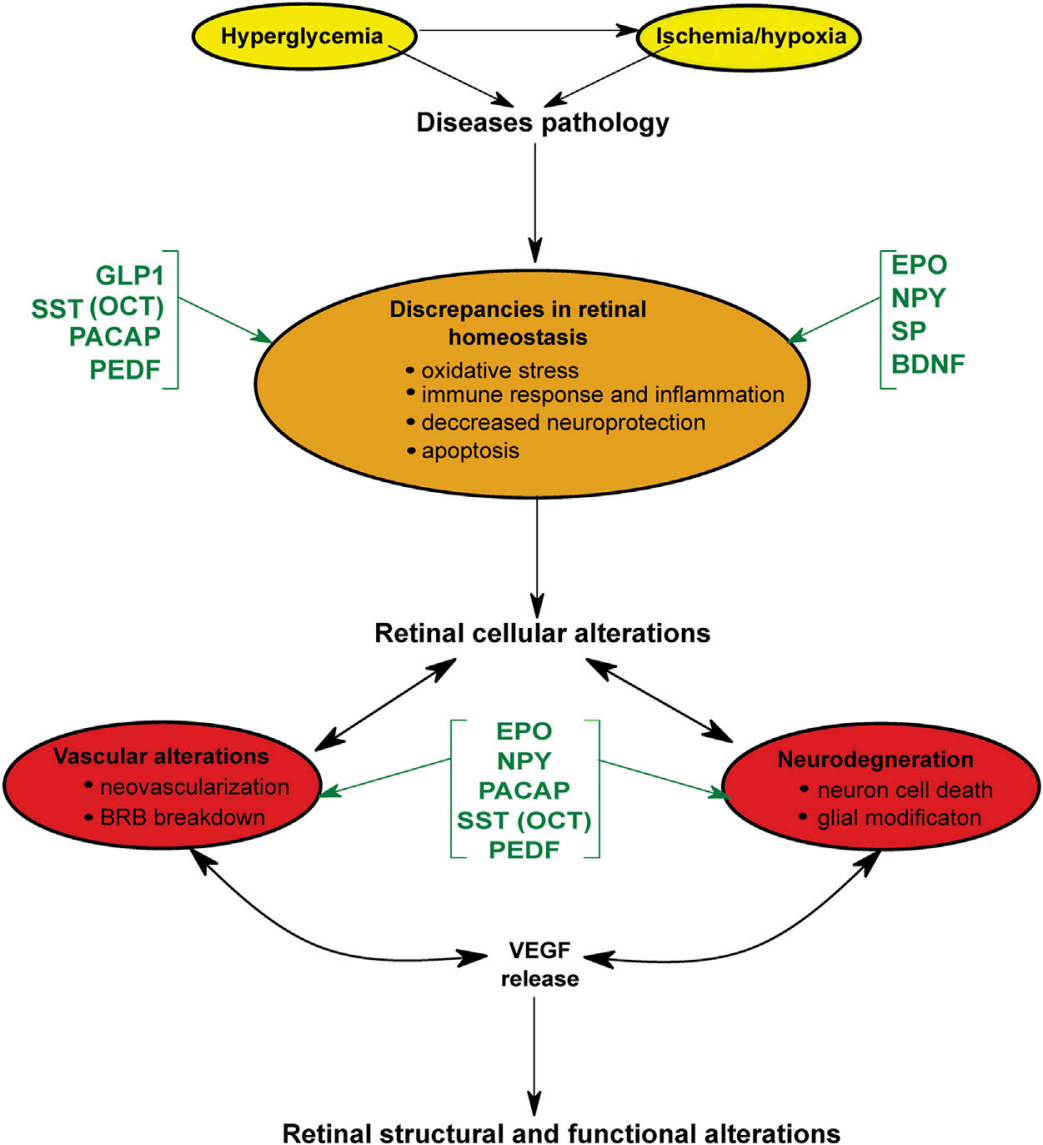
Schematic representation of the curative role and point of action of different neuropeptides and other factors in retinal diseases. Yellow: the initial emergency situation at the onset of disease. Orange: early alterations/damages occurring at cellular level. Red: irreversible damage that leads to pathological structural and functional alterations in the retina. Abbreviations: GLP-1, glucagon-like peptide-1; SST, somatostatin; OCT, octreotide; PACAP, pituitary adenylate cyclase-activating polypeptide; PEDF, Pigment epithelium-derived factor; EPO, erythropoietin; NPY, neuropeptide Y; SP, substance P; BDNF, brain-derived neurotrophic factor; VEGF, Vascular endothelial growth factor.

As part of the central nervous system, the retina is especially vulnerable to pathologies leading to neuronal cell loss and tissue damage. Diseases affecting the structural integrity of the retina are known to cause irreparable damage, leading to the slow degradation of visual acuity and in certain cases, eventual blindness. Over the years, various NPs have been shown to exert a certain degree of neuroprotective capacity in diabetic and ischemic retinas (Table 2), leading to a discovery and development of new therapeutic agents. These molecules are not only able to mitigate severe and irreversible damage (cell death and uncontrolled neovascularization) but also counteract early manifestations of pathological disturbances, including oxidative stress and inflammation (Figure 1). Considering the role NPs play in alleviating (or reinforcing) disease pathophysiology, we can summarize the conclusion of the current paper in four concise points.

### 5.1 Neuropeptide Homeostasis Needs to Be Maintained for a Healthy Retina

While the normal function of NPs is often multifaceted (e.g., SST) or elusive and hard to grasp (e.g., PACAP), it is undeniable that an upset in NP production can lead to serious consequences for retinal health (Table 2; Figure 1). The fact that NP effects are often only measurable when a) the substance is fed into the system in excessive amounts or b) normal retinal function is disturbed suggests that these molecules are designed to work on a small scale doing the “housekeeping” and maintaining everyday retinal homeostasis. Considering how protective peptide levels are significantly decreased in retinas affected by DR (see Section 4.1.1) and how multiple NPs have been more or less effective in mitigating pathological damage, it is safe to assume that these molecules are not just complementary but essential for retinal function. It is worth noting, however, that the cellular localization of NPs and their receptors is yet to be fully mapped in all relevant species.

### 5.2 Different Neuropeptides Might Require Different Methods of Application

Another important issue has been the selection of the proper delivery system for experiments aiming to better understand the effects of NPs: any systemic delivery is hindered as long as the BRB is intact. One of the successful solutions to circumvent molecule size constraints has been the use of OCT instead of SST. Regardless, the widespread expression of NPs and NPRs across the body warrants extra care in the selective use of NPs for their retinoprotective ability and topical administration is generally preferred in practice. Eye drops can potentially be utilized as a non-invasive option but this method also suffers from the presence of barriers (Werling et al., 2017). However, eye drops have already been successfully implemented as a method of delivery, including the usage of PACAP against glaucoma (Szabo et al., 2021) and SST against DR (Hernández et al., 2013). While intraocular injections offer a direct solution to the problem of permeability, their invasive nature makes them dangerous as the adverse effects of repeated injections (inflammation, retinal detachment) can have severe consequences on retinal health. To circumvent the necessity for repeated injections, nanoparticle mediated delivery has been tested with promising results for OCT application (Amato et al., 2018). In addition, a number of other methods are being considered, including biodegradable implants (Mandal et al., 2018). It is important to note that NPs used for experimental or therapeutic purposes might also need to be stabilized against degradation and enzymatic breakdown (Mandal et al., 2018; Kovacs et al., 2021), meaning that choosing the method of delivery is crucial for any curative research.

### 5.3 Not All Neuropeptides Possess the Same Therapeutic Potential

All NPs contribute to retinal health, just not in the same manner and not to the same degree. While most NPs appear to possess neuroprotective capabilities in addition to a variety of other functions (see Table 1 and Figure 1), some of these substances are more effective in combating pathological changes than others. GLP-1 and its analogs, for example, convey a much more widespread effect compared to the other major NPs. Similar to GLP-1 and other GLP-1 receptor agonists, the SST analog octreotide has also proven to be a versatile tool in mitigating damage to the retina under pathological conditions, just like PACAP (Table 2). At this point in time, it is probably safe to state that any latent therapeutic potential for all known NPs have been adequately researched. However, even the most effective candidates have proven to be insufficient in combating DR and ischemia on their own. To our surprise, there has been little interest so far in combination treatments utilizing two or more NPs, even though the therapeutic potential of already existing drugs and neuroprotective substances could potentially be increased with the right combination. We strongly believe that it is time for NP research to take the next step and move on from trying to find one ideal substance and focus on exploring the potential in neuroprotective synergies.

### 5.4 Transgenic Models Are Still Underrepresented in the Research of Neuropeptides

Another conclusion of this study is that transgenic models are still not being exploited to their full potential in the research of NP effect on retinal health. Combined with the fact that no NP alone has been able to convey full protection against the deleterious effects of DR or ischemia, targeted genetic modification and controlled simultaneous NP overexpression could offer new prospects in scientific research. One option would be the generation of conditional knock-ins connected to NP production, in which different well-known pathological mediators (e.g., VEGF) could act as the driving factor behind the transcription process of NP genes. Considering their widespread protective effects, OCT or PACAP seem to be the most ideal candidates for this job. The second, somewhat different approach could be the application of different enhancer sequences to the transgenic NP construct. At the same time, it is important to mention that NPs with retinoprotective capacity are not only expressed in the retina and ensuring cell and tissue specificity in any construct (e.g., tissue specificity of the promoter) ought to be a major concern.

## Glossary

AC: amacrine cell
ACE: angiotensin converting enzyme
ACE2: angiotensin converting enzyme 2
Ang II: angiotensin II
ARB: angiotensin receptor blocker
ATR: angiotensin receptor
AT1R: angiotensin II type 1 receptor
AT2R: angiotensin II type 2 receptor
BC: bipolar cell
BDNF: brain-derived neurotrophic factor
BRB: blood-retina-barrier
cAMP: cyclic adenosine monophosphate
CAO: cerebral artery occlusion
cGMP: cyclic guaninosine monophosphate
dAC: displaced amacrine cell
DM: diabetes mellitus
DR: diabetic retinopathy
E4: exendin-4
eNOS: endothelial nitric oxid synthase
EPO: erythropoietin
ERK1/2: extracellular signal-regulated kinase
GLP-1: glucagon-like peptide-1
GLP-1R: glucagon-like peptide-1 receptor
GLP-1RA: glucagon-like peptide-1 receptor agonist
GM: genetically modified
HC: horizontal cell
ICAM: intercellular adhesion molecule
IL-1β: interleukin 1 beta
IOP: intraocular pressure
IP3: inositol trisphosphate
IRI: ischemia reperfusion injury
JAK: janus kinase
KO: knockout
MAPK: mitogen activated protein kinase
mTOR: target of rapamycin
NF-κB: nuclear factor kappa B
NK: neurokinin
NO: nitric oxide
NP: neuropeptide
NPY: neuropeptide Y
OCT: octreotide
PACAP: pituitary adenylyl cyclase-activating polypeptide
PE: pigment epithelium
PEDF: pigment epithelium derived factor
PI3K: phosphoinositide 3-kinase
PKA: protein kinase A
PKC: protein kinase C
PLC: phospholipase C
PR: photoreceptor
RAS: renin-angiotensin system
RGC: retinal ganglion cell
ROS: reactive oxygen species
RPE: retinal pigment epithelium
SIRT: sirtuin
SP: substance P
SST: somatostatin
STAT: signal transducer and activator of transcription
STZ: streptozotocin
TrkB: tropomyosin receptor kinase B
VEGF: vascular endothelial growth factor
VIP: vasoactive intestinal polypeptide
