# Supplementary material for: Relevance of Peptide Homeostasis in Metabolic Retinal Degenerative Disorders: Curative Potential in Genetically Modified Mice
Source: Front Pharmacol. 2022 Jan 13;12:808315. doi: 10.3389/fphar.2021.808315 (PMC8793341; doi:10.3389/fphar.2021.808315)
Supplement: Supplementary file 1 [file Table1.DOCX]

***Supplementary Material***

# Table S1. List of used transgenic alleles in mouse models of retinal diseases (source: Mouse Genome Informatics, <http://www.informatics.jax.org/>). Articles in the supplementary material are referenced through their PubMed identifiers (PMID).

| **Diseases** | **Allele Symbol** | **Reference** |
| --- | --- | --- |
| **retinitis pigmentosa** | *Tg(Rho-Arl3*Q71L)#Visu* | PMID: 26936825 |
|  | *Tg(CAG-Rpgr)mRDefWrght* | PMID: 21546531 |
|  | *Tg(Rho)1Wbae* | PMID: 8516292 |
|  | *Tg(RHO*P347S)A1Tili* | PMID: 29242588,PMID: 22809998 |
|  | *Tg(RHO-P23H)DTpd* | PMID: 1418997 |
|  | *Tg(BEST1-rtTA,tetO-cre)1Yzl* | PMID: 32245241 |
|  | *Tg(Chx10-EGFP/cre,-ALPP)2Clc* | PMID: 23001562,PMID: 24339791 |
|  | *Tg(Crx-cre)1Tfur* | PMID: 29100828,PMID: 24493795 |
| **retinoblastoma** | *Tg(Pax6-TAg)1796Hur* | PMID: 22562503 |
|  | *Tg(Rbp3-SV40)IT-2Jjw* | PMID: 8112979 |
|  | *Tg(TagRb)1Plm* | PMID: 1689463 |
|  | *Tg(Nes-cre)1Atp* | PMID: 15231717 |
|  | *Tg(Pax6-cre,GFP)2Pgr* | PMID: 11301001,PMID: 17235288 |
| **age related macular degeneration** | *Tg(APOB)1102Sgy* | PMID: 19450445 |
|  | *Tg(BEST1-cre)1Jdun* | PMID: 25257511 |
| **retinal cancer** | *Tg(Tyrp1-TAg)3434Bee* | PMID: 11896616 |
| **cone-rod dystrophy** | *Tg(Crx-AIPL1*P351)#Visu* | PMID: 25274777 |
|  | *Tg(Rho-GUCY2D*R838S)362Amd* | PMID: 27703005 |
|  | *Tg(Rho-GUCA1A*Y99C)L53Amd* | PMID: 22042849 |
|  | *Tg(Rho-GUCY2D*R838S)379Amd* | PMID: 27703005 |
| **Stargardt disease** | *Tg(RBP3-ELOVL4*)1Kzh* | PMID: 15749821 |
|  | *Tg(RBP3-ELOVL4*)3Kzh* | PMID: 15749821 |
| **Leber congenital amaurosis** | *Tg(Chx10-EGFP/cre,-ALPP)2Clc* | PMID: 23001562,PMID: 24339791 |
|  | *Tg(Crx-cre)1Tfur* | PMID: 29100828,PMID: 24493795 |
|  | *Tg(rx3-icre)1Mjam* | PMID: 22398208 |
| **retinal degeneration** | *Tg(Rho-icre)1Ck* | PMID: 29659833 |
| **X-linked cone-rod dystrophy** | *Tg(CMV-Rpgr)1Tili* | PMID: 14691151 |
